# Eserine1Read before the semi-annual meeting of the Pennsylvania State Veterinary Medical Association, Pittsburg, September, 1898.

**Published:** 1899-08

**Authors:** E. E. Bittles

**Affiliations:** New Castle, Pennsylvania


### ESERINE.[1]

By Dr. E. E. Bittles,
NEW CASTLE, PENNSYLVANIA.

Veterinarians differ greatly as to the action and use of eserine, some claiming it worthless, others a great cure-all. I think that this great difference of opinion is due to the fact that some never try it until they are positive the patient must succumb; that some giving too large and others too small doses; faulty preparations, etc.

Of the various preparations of eserine the sulphate is preferable, and should always be obtained in sealed glass tubes of two grains each, as they are easily carried and one tube is sufficient for any one case (seldom using more than one-half of it) as it is not advisable to repeat the dose in less than three or four hours.

In preparing the solution use one drachm of distilled water to one grain of eserine. One drachm of the above solution used hypodermically is a safe and usually effective dose for an animal of one thousand pounds. The solution will keep for several days, if kept in a well-stoppered bottle in a dark place.

I have obtained the best results from its use in those cases of acute indigestion where the bowels and stomach are greatly distended with gas; no audible intestinal sound; the entire tract temporarily paralyzed from the great strain caused by the pressure of the gases, and space at a premium in those parts, the stomach being full to overflowing. Bearing this in mind, and drenches being out of the question, we resort to a more modern treatment, and give hypodermically one grain of

[1] Read before the semi-annual meeting of the Pennsylvania State Veterinary Medical Association, Pittsburg, September, 1898.

eserine, which will increase the peristaltic action of the bowels and carry off the gases. To prevent further formation of gas I have never found anything better than one ounce of pulverized ginger; one drachm of beechwood creosote, two drachms of chloroform, given in capsules, repeating the dose in half an hour if necessary. If the breathing is labored, and there is danger of suffocation, would use the trocar.

In all stages of bowel-trouble caused by the formation of gas, I have never found anything to equal eserine. Barium chloride has about the same action, but it is more difficult to administer on account of its being very irritable following its use. Also, in some cases, we have paralysis, causing death. In cases of subacute indigestion, impaction, constipation, or any of those conditions of torpidity where the symptoms are not urgent, I prefer aloes, and, if that does not give relief in twelve to fifteen hours, use eserine, which will cause the aloetic purge to work off nicely. I find eserine does not give relief in those cases in the first stages.

My experience with eserine in parturient apoplexy has been, it helped them along to the place where so many of these cases go,—the boneyard.

---

Philadelphia health authorities had a public demonstration of the importance of bovine tuberculosis in a herd early in August, where physical examination revealed two suspected cases, and a tuberculin-test detected eighteen out of twenty-one. A public autopsy revealed the correctness of the test, and laid bare lesions of an extensive character in many that the physical appearance of the animals did not indicate.

Society owes to the horse a debt of gratitude a thousand times greater than it does to thousands of men who abuse him. He has ministered to progress; has made social intercourse possible, when otherwise it would have been slow and occasional, or altogether impossible; he has virtually extended the strength of man, augmented his speed, doubled his time, decreased his burdens, and, becoming his slave, has relieved him from drudgery and made him free. For love's sake, for the sake of social life, for eminent moral reasons, the horse deserves to be bred, trained, and cared for with scrupulous care. The teaching of men how to do it has been left too long to men who look upon the horse as an instrument of gambling gains, or of mere physical pleasure.

